# Vacuum Dynamics as an Alternative Method for Detection of Bimodal Milk Ejection in Dairy Cows

**DOI:** 10.3390/ani11071860

**Published:** 2021-06-23

**Authors:** Matthias Wieland, Christina Marie Geary, Gloria Gioia, Kerry Lynn Case, Paolo Moroni, Anja Sipka

**Affiliations:** 1 Department of Population Medicine and Diagnostic Sciences, Cornell University, Ithaca, NY 14853, USA; cmg268@cornell.edu (C.M.G.); gg363@cornell.edu (G.G.); klc22@cornell.edu (K.L.C.); pm389@cornell.edu (P.M.); ass233@cornell.edu (A.S.); 2 Dipartimento di Medicina Veterinaria, Università degli Studi di Milano, Via dell’Università, 6, 26900 Lodi, Italy

**Keywords:** bovine, bimodality, milk flow, teat end vacuum

## Abstract

**Simple Summary:**

We investigated the relationship between vacuum dynamics and milk flow curve characteristics using portable vacuum and milk flow recording devices, respectively, for the assessment of bimodal milk flow curves in dairy cows. For this purpose, we analyzed 241 vacuum and milk flow curve recordings that we collected concomitantly during eight milking center evaluations on five New York dairy farms. We found that vacuum dynamics could be a suitable measure to assess bimodal milk flow curves in dairy cows.

**Abstract:**

The primary objective of our study was to assess the ability of a vacuum recorder to detect the presence of bimodal milk flow curves in dairy cows compared with a portable milk flow meter. In a cross-sectional study, 241 individual cow milking observations were analyzed. We simultaneously collected (1) individual cow vacuum events during milking using portable vacuum recorders, and (2) individual cow milk flow curves by attaching a portable milk flow meter to the same milking unit. Presence of bimodality was assessed with the vacuum recorder visually (BIM_VA_) and with the gold standard method of a milk flow meter through automatic detection (BIM_LA_). Kappa statistics revealed moderate agreement between BIM_VA_ and BIM_LA_ [κ, 95% confidence intervals (95% CI) = 0.59 (0.46–0.71)]. Diagnostic test statistics for BIM_VA_ for detection of bimodality indicated moderate performance for sensitivity [0.65 (0.52–0.76)] and positive predictive value [0.71 (0.58–0.82)] and high values for specificity [0.92 (0.87–0.95)] and negative predictive value [0.93 (0.84–0.93)]. We conclude that milking vacuum dynamics are a suitable measure to assess bimodal milk flow curves in dairy cows.

## 1. Introduction

The analysis of milk flow dynamics provides valuable information for improving milking efficiency and enhancing udder health on dairy operations [1]. Among the numerous measures of milk flow dynamics, bimodality has been used extensively to assess the quality of pre-milking teat stimulation. A bimodal milk flow curve is defined when an increasing milk flow rate is followed by a decreasing flow rate during the first 2 min of milking [2]. It is due to removal of the cisternal milk fraction before the alveolar milk reaches the gland cistern [3] or when the milk flow out of the teat canal exceeds the milk flow to the teat cistern, a phenomenon that Rasmussen defined as overmilking [4]. Bimodality is a result of insufficient stimulation before milking [5,6] and has been associated with decreased milking efficiency [7], reduced milk yield [8,9,10], and impaired udder health [10,11].

Traditionally, milk flow dynamics have been assessed with either portable or stationary electronic milk meters that measure continuous milk flow. Among the portable milk meters, the Lactocorder (WMB AG, Balgach, Switzerland) has gained significant popularity among veterinarians and milk quality consultants worldwide. Data from the Lactocorder have been established as measures of milking machine settings [5], pre-milking teat stimulation [6], and genetic evaluations of milk flow traits of individual cows [1,12,13,14]. Indeed, the Lactocorder has been considered the gold standard for field assessment of milk flow dynamics in dairy cows over the last few decades.

Due to an inverse linear relationship between vacuum and milk flow [6,15,16], instruments that record vacuum in the milking unit may be valuable tools to assess milk flow dynamics during individual cow milking observations [8]. VaDia vacuum recorders (Biocontrol, Rakkestad, Norway) facilitate synchronous vacuum measurements in four channels. The device can be attached to the cluster without a major impact on handling ease of the milking unit during milking. One operator can attach and oversee numerous recorders in a milking parlor concomitantly. This allows collection of data from large numbers of cows at different positions within milking strings [8]. In recent studies, VaDia vacuum recorders have been used to further the understanding of the relationship between vacuum dynamics, milk flow, and teat end condition [17], between milking-time testing and udder health status [18], and between herd-level variables and overmilking [19], as well as delayed milk ejection [20]. However, to our knowledge, no information is available regarding the association between changes in milking vacuum dynamics as assessed with the VaDia vacuum recorder and the presence of bimodality as assessed with the Lactocorder. Knowledge about this relationship would offer unique opportunities to help the industry develop milk harvesting strategies that result in improved milking efficiency, milk yield, and udder health. Therefore, our primary objective was to investigate if the VaDia vacuum recorder can be used to assess bimodality as determined with the Lactocorder. We hypothesized that vacuum dynamics measured in the short milk tube and mouthpiece chamber with the VaDia vacuum recorder could serve to identify bimodality as assessed with the Lactocorder.

In recent studies using the VaDia vacuum recorder, researchers employed the “let down time” (LDT) to study which herd-level variables were associated with delayed milk ejection [20] and determine the association of delayed milk ejection on milk yield [8]. The LDT can be calculated with the adjacent software program and is defined as the time interval between start of milking (cluster attachment) and start of peak flow period. It also has been referred to as the time interval between the start of milking and the start of the incline phase of milk flow [8,20] and the time delay from cluster attachment to true milk ejection [21]. These previous studies provided invaluable information that enhance our understanding of the milk ejection in dairy cows and help improve the milk harvesting process. However, the relationship between LDT and bimodality as assessed with the Lactocorder has not been established by rigorous methods. Our secondary objective therefore was to study if LDT can be used as a proxy for bimodal milk ejection as assessed with the Lactocorder device. We hypothesized that an association existed between LDT and bimodality, as assessed with the Lactocorder.

Bimodality has been associated with decreased milking efficiency [7] and reported to cause production loss [10]. Previous researchers studying the effect of different pre-milking stimulation regimens on bimodality and machine on time reported an increased frequency of bimodality together with increased machine on time in cows who received no or less stimulation before milking [7,22,23]. However, none of these studies directly investigated the association between bimodality (as independent variable) and machine on time (as dependent variable). In a recent study, Erskine et al. [8] employed the LDT to assess delayed milk ejection and found a negative association between delayed milk ejection and milk yield. The third objective of our study was to add to the existing body of literature and investigate the associations of bimodality and LDT on milk yield and machine on time. Specifically, we set out to determine if bimodality as assessed with the VaDia vacuum recorder and the Lactocorder, respectively, as well as LDT can help explain the variabilities in milk yield and machine on time.

Vacuum-induced mechanical forces during machine milking can cause congestion and edema of the teat tissue [24]. These machine milking-induced short-term changes (STC) are thought to decrease the teats’ defense mechanisms [25,26] and were associated with increased risk of new intramammary infection [27]. Additionally, short-term changes are considered to diminish animal well-being [28]. Due to the inverse relationship between vacuum and milk flow [6,15,16], bimodality can increase the vacuum-induced mechanical forces to the teats during the transient period of low or zero milk flow. Additionally, low milk flow can lead to a poor seal between the teat barrel and the milking liner wall, allowing the milking vacuum to penetrate into the mouthpiece chamber [15]. The subsequent increase in mouthpiece chamber vacuum may lead to increased teat congestion [29]. Our fourth objective therefore was to investigate the association of bimodality and LDT on STC. We hypothesized that bimodality and increased LDT would increase the risk of STC.

## 2. Materials and Methods

This cross-sectional study was conducted between May 2019 and August 2020 on 5 commercial dairy farms in New York State, USA. All farms belonged to the client base of Quality Milk Production Services of Cornell University (QMPS; Ithaca, NY, USA) and were selected based on their willingness to participate in the study. Farm 1 milked 1300 cows, farms 2 and 3 milked 1400 cows, farm 4 milked 4000, and farm 5 milked 1350 cows. Holstein cows were housed in free-stall pens with either concrete stalls covered with mattresses and bedded with wastepaper-pulp (farm 1), manure solids (farm 4, 3/10 pens), deep-bedded stalls with fresh sand (farm 2) or manure solids [farms 3, 4 (7/10 pens), and 5]. All cows were milked 3 times daily in parallel (farms 1, 2, 3, and 5) or rotary (farm 4) milking parlors. Milking liners used were: Impulse IP4-LM-SB (Milkrite, Melksham, UK; farms 1 and 5); Clover 12R+ (DeLaval International AB, Tumba, Sweden; farm 2); MR-T4-51 (Conewango, Products Corporation, Falconer, NY, USA; farm 3); and LS-01 NC (DeLaval International AB; farm 4). The milking routine on farms 1, 2, 3, and 5 was based on a territorial routine, where milking technicians are assigned to milking stalls on both sides of the parlor and only operate the milking units assigned to them. The parallel parlor premilking udder preparation consisted of (1) predipping teats with a teat disinfectant, (2) forestripping of teats, (3) drying and cleaning of teats with a clean cloth towel, and (4) attachment and alignment of the milking unit. The rotary parlor at farm 4 was operated by 4 milking technicians who were assigned to 4 different positions. Four teat spray robots (TSR, DeLaval International AB; 2 at the parlor entrance and 2 at the parlor exit) were installed for pre- and postmilking teat dip application. Here, premilking udder preparation consisted of (1) application of premilking teat disinfectant, (2) drying and cleaning of teats with a clean cloth towel [positions 1 (focus on teat barrel) and 2 (focus on teat end)], and (3) attachment and alignment of the milking unit (position 3). Position 4 was to monitor milking liner slips, unit fall-offs, and unit kick-offs and realign or reattach the milking unit accordingly. All farms used a dairy management software program (Dairy Comp 305, Valley Agricultural Software, Tulare, CA, USA) to maintain herd data, as well as monthly (farms 2, 3, and 5) or bimonthly (farms 1 and 3) DHIA services including the individual-cow SCC option. To ensure that vacuum stability of the milking system was not a variable, we evaluated the system vacuum capacity with a “unit fall-off test” during each visit according to the guidelines outlined by the National Mastitis Council [30].

### 2.1. Data Acquisition

Data were collected in conjunction with milking center evaluations provided by QMPS during 8 separate visits (farms 1 and 2, 1 visit; farms 3–5, 2 visits). Table 1 shows baseline characteristics of milking parlor and equipment, machine settings, and milking routine timings assessed during each visit. We used 2 VaDia vacuum recorders and 2 Lactocorder devices throughout the study. We positioned the recording devices such that the first and last cows of 2 territories (territory = set of cows prepped by 1 milking technician) were recorded. In the rotary parlor, we placed the devices 5 stalls apart to facilitate data collection. Thus, cows were enrolled randomly based on their order of entrance into the milking parlor.

### 2.2. Vacuum and Milk Flow Recordings

**Figure 1:**
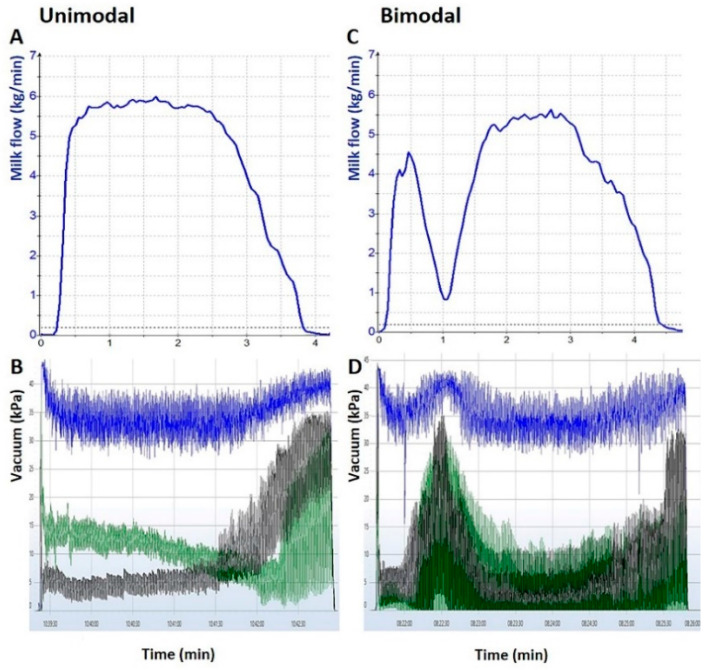
Comparison of unimodal (**A**,**B**) and bimodal (**C**,**D**) milk ejection as assessed with the Lactocorder (WMB AG, Balgach, Switzerland; **A**,**C**) and the VaDia (Biocontrol, Rakkestad, Norway; **B**,**D**). The Lactocorder graphs show milk flow as milk (kg/min) and the VaDia graphs depict the milking as vacuum (kPa) over time (min). (**B**,**D**) Channel 2 (blue line) represents the vacuum measured in the short milk tube. Channels 3 and 4 (green and black lines) represent the vacuum measured in the right hind and left front mouthpiece chambers, respectively.

Individual cow vacuum events during milking were collected using VaDia vacuum recorders as previously described [20]. Briefly, 4 vacuum channels were used for each individual cow observation by attaching 2.4-mm (internal diameter) silicon tubing to the following positions on the milking unit: (1) channel 1, short pulsation tube of right hind teat cup to record pulsation vacuum; (2) channel 2, short milk tube of the right hind teat cup to record teat end vacuum; (3) channel 3, liner mouthpiece of the right hind teat cup to record mouthpiece chamber vacuum; (4) channel 4, liner mouthpiece of the left front teat cup to record vacuum in the mouthpiece chamber. Vacuum recordings were reviewed and analyzed with the VaDia Suite software program (version 1.13.0.860; Biocontrol, Rakkestad, Norway) by 1 investigator (MW) who was blinded to the results of the Lactocorder recordings. We determined key events such as start of milking, start of peak flow period, start of cyclic vacuum fluctuations, start of overmilking, start of take-off, and end of milking by visual assessment rather than the automated detection of the software program as previously described [8,9,19,20,21]. Subsequently, the following parameters were calculated with the software program: machine on time, the time interval between start and end of milking; LDT, time interval between start of milking and start of peak flow period; overmilking period, time interval between start of overmilking and start of take-off; average mouthpiece chamber vacuum, average vacuum of all data points of the mouthpiece chamber during peak flow period and overmilking period; and average cyclic vacuum fluctuations, overall average of average short milk tube vacuum calculations from ten pulsation cycles 60 s after the start of the peak flow period. In addition, we evaluated the presence or absence of bimodality (BIM_VA_). A BIM_VA_ was present when mouthpiece chamber vacuum and teat end vacuum decreased after the start of milking but then increased markedly as defined by Erskine et al. [8]. Figure 1 depicts 2 milking observations with unimodal (A and B) and bimodal (C and D) milk ejection.

Individual cow milk flow curves from the same milking observations were assessed simultaneously by attaching the Lactocorder to the same milking unit. Milk flow curves were reviewed and analyzed by 1 investigator (CMG) with the adjunct software program (LactoPro, WMB AG, Balgach, Switzerland) who was blinded to results of the VaDia recordings. Presence of a bimodal milk flow curve (BIM_LA_) was automatically assessed by the software program and detected during the first 96 s after milk flow exceeded 0.5 kg/min if 1 of the following conditions were met: (1) milk flow reflux (i.e., decrease in milk flow rate following the initial increase) >0.2 kg/min, (2) milk flow reflux >0.1 kg/min and the interruption of the incline phase ≥14 s. In both cases, the milk reflux had to amount to ≥16% of the maximum milk flow but a minimum of 0.5 kg/min if the milk flow of the first peak reached or exceeded 80% of the maximum milk flow. Additionally, we documented the integrity of milk flow curves and obtained the individual cow milk yield (kg) from the adjunct software program.

### 2.3. Nonlactating Quarter and Teat Tissue Condition

Two trained investigators (MW and CMG) assessed the presence of a nonlactating quarter and post-milking teat tissue condition. The presence of a nonlactating quarter was visually assessed during milking and affirmed present if 1 teat cup was not attached to the respective teat. Machine milking-induced short-term changes to the teat tissue condition were assessed visually and through palpation according to the scoring system described by Hillerton et al. [31]. Briefly, within 60 s after unit detachment, we classified the color of the teat skin as normal (score 1), red (score 2), or blue (score 3). The condition of the teat base was classified as no visible mark present (score 1), visible mark present (score 2), or palpable swelling (score 3). The evaluation of consistency of the teat end was scored as soft (score 1), firm (score 2), or wedging present (score 3). The presence of a STC was considered if the color of the teat skin was score 3, or the condition of the teat-base score was 3, or the consistency at the teat-end score was ≥2 for 1 or more teats; STCs were absent otherwise.

### 2.4. Sample Size Calculation

We elected to base the sample size calculation on the kappa statistics employed to assess the agreement in detecting bimodality between the VaDia vacuum recorder (BIM_VA_) and the Lactocorder device (BIM_LA_). The calculation was performed with the ‘irr’ package [32] in R Statistical Software [33] and based on the following assumptions. A probability that the VaDia vacuum recorder would detect bimodality of 15%, a probability that the Lactocorder would record a bimodal milk flow curve of 15%, a true Cohen’s kappa statistic of 0.7, a value of kappa under the null hypothesis of 0.4, and a two-sided test. We applied a power of 0.95 and an alpha-level of 0.05. The resulting sample size of 190 measurements was then inflated by a factor of 0.5 due to an assumed attrition rate of 50%, resulting in a pre-exclusion sample size of 285 individual cow milking observations.

### 2.5. Descriptive Statistics

Data were maintained in Excel (2016 version, Microsoft Corp., Redmond, WA, USA) and JMP (version 14, SAS Institute Inc., Cary, NC, USA). Prior to statistical analyses, we investigated the data for missing and erroneous values. Observations with missing VaDia or Lactocorder data, incomplete (i.e., milk flow graph cut-off) or erroneous (i.e., milk flow curves depicting ‘static milk’) milk flow curves were excluded. To give each observation equal weight in the analyses, we included only the first observation from each cow. Additionally, we excluded observations from cows with a non-lactating quarter.

Differences in milk yield, machine on time, let down time, overmilking period, average mouthpiece chamber vacuum, and average cyclic vacuum fluctuations of milking observations with and without BIM_VA_ and BIM_LA_, respectively, were assessed using Student’s *t*-test for variables with equal variances and with the Welch’s test for variables with unequal variances as assessed with the Levene’s test. Pearson chi-square tests were used to assess differences in STC between milking observations with and without BIM_VA_ and BIM_LA_, respectively.

### 2.6. Comparison of VaDia and Lactocorder Devices

To test the hypothesis that the VaDia vacuum recorder could serve to identify bimodality as assessed with the Lactocorder, we used 2 different approaches. In the first step, we calculated kappa (κ) statistics to assess the agreement beyond chance between the 2 binary variables BIM_VA_ and BIM_LA_ using the package ‘DescTools’ [34] in R. We interpreted the κ value according to Landis and Koch [35] as follows: a κ value of <0.21 was interpreted as poor agreement, 0.21–0.40 as fair, 0.41–0.60 as moderate, 0.61–0.80 as good, and 0.81–1.00 as very good agreement. Second, we calculated sensitivity, specificity, and positive and negative predictive values to evaluate the diagnostic performance of BIM_VA_ in detecting bimodality using BIM_LA_ as the gold standard as described by Dohoo et al. [36]. The 95% confidence intervals (95% CI) for sensitivity, specificity, and positive and negative predictive values were calculated as *a* ± 1.96 × standard error, where standard error = $\sqrt{a\left(1-a\right)/n}$ with *a* being the test proportion and *n* being the sample size. Values ≤0.60 were interpreted as low, 0.61–0.80 as moderate, and values >0.80 as high according to Royster et al. [37].

To determine whether LDT could serve as a proxy for bimodal milk ejection as assessed with the Lactocorder device, we fitted a generalized linear mixed model with a logit link and a binomial distribution using the ‘lme4’ package [38] in R. The binary variable BIM_LA_ was included as dependent variable and the continuous variable LDT as independent variable. Parity (1st, 2nd, and ≥3rd lactation) and stage of lactation (≤100, 101–200, >200 DIM) were included as covariates and forced into the model. Farm was included as a random effect to account for the clustered structure of the data.

### 2.7. Bimodality, Let Down Time, and Milking Characteristics

To determine the associations of BIM_VA_, BIM_LA_, and LDT on the milking characteristics milk yield (kg) and machine on time (s), respectively, we fitted 6 separate general linear mixed models with the ‘lme4’ package [38]. That is, for each of the 2 dependent variables (i.e., milk yield and machine on time), we fitted 3 separate models including BIM_VA_, BIM_LA_, and LDT one at a time as independent variable, respectively. The following 2 steps were consistent for all 6 models: Farm was included as random effect to account for the hierarchical nature of the data. Parity (1st, 2nd, and ≥3rd lactation) and stage of lactation (≤100, 101–200, >200 DIM) were included as covariates and forced into all models. For the dependent variable machine on time, we included milk yield as a covariate. We calculated least squares means (LSM) and 95% CI with the ‘emmeans’ package [39]. Finally, we inspected residual plots versus corresponding predicted values and examined quantile-quantile residual plots to assess if the assumptions of homoscedasticity and normality of residuals were met.

### 2.8. Bimodality, Let Down Time, and Teat Tissue Condition

To test the associations of BIM_VA_, BIM_LA_, and LDT on STC, we built 3 separate generalized linear mixed models with a logit link and a binomial distribution with the ‘lme4’ package [38]. The following 2 steps were consistent for all 3 models: Farm was included as a random effect in all models to account for the clustered structure of the data. Parity (1st, 2nd, and ≥3rd lactation), stage of lactation (≤100, 101–200, >200 DIM), and machine on time were included as covariates and forced into all models. Further, BIM_VA_, BIM_LA_, and LDT were included one at a time.

## 3. Results

We collected data from a total of 331 individual cow milking observations. One observation (0.3%) from a cow that was recorded twice and data from 29 (9%) cows with a non-lactating quarter were excluded. Among the 301 remaining observations, 54 (18%) observations had no VaDia recordings, 4 (1%) Lactocorder milk flow curves were erroneous, and 2 (1%) observations had both no VaDia data and erroneous Lactocorder milk flow curves. This resulted in 241 individual cow milking observations with complete VaDia and Lactocorder data that were available for further analyses.

### 3.1. Study Population

Cows were in their first (104, 43%), second (75, 31%), and third or greater lactation (62, 26%), between 2 to 384 DIM [mean (SD) 148 ± 97], and had a median SCC at their last test day of 38,000 cells/mL [mean (SD) 135,200 ± 287,000]. The average (mean ± SD) milk yield as recorded with the Lactocorder was 14.7 ± 4.1 kg. The average (mean ± SD) values for machine on time, LDT, and overmilking period, respectively, were 270 ± 84, 32 ± 20, and 48 ± 44 s. The mean ± SD average cyclic vacuum fluctuations and mouthpiece chamber vacuum, respectively, were 35.2 ± 3.6 and 16.3 ± 8.4 kPa. Presence of BIM_VA_ was documented in 52 (22%) cases and presence of BIM_LA_ was recorded in 57 (24%) milking observations. We documented presence of STC in 127 (53%) cases. Table 2 depicts descriptive statistics stratified by farm.

### 3.2. Comparison of VaDia and Lactocorder Devices

Table 3 shows the frequency distribution of BIM_VA_ and BIM_LA_ in a 2 × 2 table. The κ value and 95% CI for the agreement between BIM_VA_ and BIM_LA_ was 0.59 (0.46–0.71) indicating moderate agreement. Using BIM_LA_ as the gold standard, diagnostic test statistics for BIM_VA_ for detection of bimodality were as follows: sensitivity, 0.65 (0.52–0.76); specificity, 0.92 (0.87–0.95); positive predictive value, 0.71 (0.58–0.82); and negative predictive value, 0.93 (0.84–0.93).

Table 4 summarizes mean values (±SD) of LDT stratified by the presence of bimodality as assessed with the VaDia vacuum recorder (BIM_VA_) and the Lactocorder (BIM_LA_), respectively. The generalized linear mixed model revealed an association between LDT and BIM_LA_ (*p* < 0.0001). Controlling for the effects of parity (*p* = 0.03) and stage of lactation (*p* = 0.14) the odds of BIM_LA_ increased by 87% for every 10-s increase in LDT [odds ratio (OR), 95% CI = 1.87 (1.56–2.25)].

### 3.3. Bimodality, Let Down Time, and Milking Characteristics

Table 4 shows milk yield and machine on time for individual cow milking observations with and without bimodality as assessed with the VaDia vacuum recorder (BIM_VA_) and the Lactocorder (BIM_LA_), respectively. For the dependent variable milk yield, the model including BIM_LA_ revealed an association with milk yield (*p* = 0.02). Controlling for the effect of parity (*p* < 0.0001) and stage of lactation (*p* < 0.0001), LSM (95% CI) for milking observations with and without BIM_LA_, respectively, were 13.9 (12.8–15.0) and 15.2 (14.3–16.2) kg. Conversely, we found no association for BIM_VA_ (*p* = 0.10) and LDT (*p* = 0.70), respectively. For the dependent variable machine on time, we found no association for BIM_VA_ (*p* = 0.36), BIM_LA_ (*p* = 0.55), and LDT (*p* = 0.38). The assumptions of homoscedasticity and normality of residuals were met for all models.

### 3.4. Bimodality, Let Down Time, and Teat Tissue Condition

The frequency distribution of STC from cow milking observations with and without bimodality as assessed with the VaDia vacuum recorder (BIM_VA_) and the Lactocorder (BIM_LA_) can be found in Table 4. We detected no association between the independent variables BIM_VA_ (*p* = 0.89), BIM_LA_ (*p* = 0.30), and LDT (*p* = 0.81) and the dependent variable STC.

## 4. Discussion

### 4.1. Comparison of VaDia and Lactocorder Devices

Our primary objective in this study was to investigate whether the VaDia vacuum recorder could serve to detect bimodality as assessed with the Lactocorder device. For this purpose, we collected, concomitantly, VaDia and Lactocorder recordings from individual cow milking observations, documented the presence of bimodality as assessed with the VaDia (BIM_VA_) and the Lactocorder (BIM_LA_) devices, respectively, and compared their agreement. Based on the κ value, we found moderate agreement between the two diagnostic tools. Further, we calculated the diagnostic test statistics of BIM_VA_ for the detection of bimodality, using BIM_LA_ as the gold standard. Our results showed moderate performance for sensitivity and positive predictive value, and high values for specificity and negative predictive value. Our data support results reported by Malmo and Mein [21] who compared the time interval from unit attachment until milk ejection using milk flow curves from Lactocorder recordings and vacuum dynamics assessed with the VaDia device (i.e., LDT) and found an association (R^2^ = 0.82) between the two diagnostic tools [21].

In the second objective, we investigated whether LDT could serve as a proxy for bimodal milk ejection as assessed with the Lactocorder device (BIM_LA_). Our results indicate an association such that for every 10-s increase in LDT the odds of BIM_LA_ increased by 87%. Our results are in accordance with those from a recent Michigan study in which researchers analyzed 3824 VaDia recordings from 64 dairy herds [20].

We conclude that both BIM_VA_ and LDT, as assessed with the VaDia vacuum recorder, can be used to estimate bimodal milk ejection in dairy cows. Due to its stand-alone ability, the VaDia device enables veterinarians and milk quality consultants to contemporarily collect additional data on, for example milking routine timings (e.g., stimulation time and preparation lag time). Such data can help provide evidence-based recommendations supporting dairy producers to establish best parlor management practices.

### 4.2. Bimodality, Let Down Time, and Milking Characteristics

The third objective was to investigate the associations between BIM_VA_, BIM_LA_, and LDT and milking characteristics. We wanted to determine if BIM_VA_, BIM_LA_, or LDT would explain some of the variability in milk yield and machine on time. For the outcome variable milk yield, we found that BIM_LA_ yielded the most parsimonious model indicating that milking observations with bimodality as determined with the Lactocorder (BIM_LA_) yielded 1.3 kg less milk compared with observations with a unimodal milk flow curve. This is in accordance with results reported by Erskine et al. [8]. The researchers from Michigan analyzed milking data from 663 Holstein cows in a 3600-cow dairy with a thrice-daily milking schedule using VaDia vacuum recorders. They found a negative association between let down time and milk yield, such that milk yield decreased as LDT increased [8]. The authors [8] attributed this to decreased intracisternal pressure of the teat during times of low milk flow and a subsequently smaller teat barrel diameter, that reportedly can result in a poor seal between the teat and the milking liner [15]. This can increase the leakage of milking vacuum into the mouthpiece chamber and lead to higher mouthpiece chamber vacuum. As described previously [15], elevated mouthpiece chamber vacuum likely causes a tighter seal between the mouthpiece lip of the milking liner and the teat base that could result in constriction of the annular ring at the teat base impeding the milk flow from the gland to the teat cistern. Further, elevated mouthpiece chamber vacuum may lead to teat barrel congestion, which has been reported to decrease the cross-sectional area of the teat canal [29]. This could hamper the milk harvesting process of individual milking observations.

In an earlier study [7], the investigators found that milking without premilking stimulation resulted in more bimodal milk flow curves but had no meaningful effect on milk yield. This is consistent with results of a recent study from our group [23]. We found that cows who received forestripping had lower odds of bimodality compared with their herdmates that were not forestripped but failed to detect differences in milk yield between the two groups [23]. Therefore, despite the apparent interrelationship between premilking stimulation, bimodality, and milk yield, the overarching question whether mitigation of bimodality through, for example, adaptation of premilking stimulation to the physiological requirements of individual cows that accounts for differences in lag time (time period from stimulation to milk ejection), size in cisternal cavity, and milk flow potential into account, would result in increased milk yield remains obscure.

The absence of an association of BIM_VA_ and LDT, respectively, on milk yield, is an unexpected finding. Based on results from Erskine et al. [8], we expected an association between LDT and milk yield such that an increase in LDT would lead to a decrease in milk yield. We believe that the discrepancy between results reported by Erskine et al. [8] and the present study can be attributed mostly to the differences in study populations.

Contrary to our expectations, we found no association of BIM_VA_, BIM_LA_, and LDT on machine on time. This contrasts with previous studies investigating the effect of different premilking stimulation regimens on bimodality and machine on time showing that an increased frequency of bimodality coincided with increased machine on time [7,22,23]. Differences in study population and analyses may account for the discrepancies between results reported in these studies and the ones reported herein.

### 4.3. Bimodality, Let Down Time, and Teat Tissue Condition

Our fourth objective was to study the association of BIM_VA_, BIM_LA_, and LDT on STC. We expected that BIM_VA_, BIM_LA_, and increased LDT would lead to an increase in the odds of STC. Our results do not support a measurable relationship between BIM_VA_, BIM_LA_, and LDT and STC. The results of the current study contrast with those of a previous study from our group showing an association between udder-level milking characteristics as assessed with electronic on-farm milk meters and STC [40]. Discrepancies in study populations and assessment technique could be variables that account for the differences observed between our previous study and this one. It is also possible that unmeasured confounding factors account for the failure to detect an association between bimodality and STC in the current study. For example, several researchers reported that bimodality is positively associated with milking speed [1,41], such that cows with a bimodal milk flow curve had higher maximum milk flow rate [41] or the frequency of bimodality increased with increasing peak milk flow rate [1]. In a recent study, we showed that milkability was highest in cows with flat teat ends [42], which in turn has been reported to decrease the risk of STC [40]. It is therefore possible that in the current study, cows with flat teat ends had a higher risk of bimodality but were less likely to exhibit STC. This may have led to bias towards the null. However, because teat-end shape was not obtained in this study, this possible explanation remains speculative.

### 4.4. Study Limitations, Practical Application, and Future Directions

Our study had some limitations that must be considered. We conducted this study on 5 dairy farms in New York with Holstein cows that are milked 3 times per day. Therefore, our results likely reflect commercial operations in this area. However, the external validity of our study may be limited to similar operations in this region. This may hamper the ability to compare results among different dairy operations. To address this limitation, we suggest refraining from benchmarking. Wallace et al. [5] states: “When evaluating parlor performance we need to determine the current status, identify problematic areas in the parlor, recommend necessary changes and finally have a means to monitor these changes”. The information obtained with the VaDia device together with additional data can be used to establish a baseline value to help monitor the progress on individual dairy operations over time.

In this study, we elected to determine key events by visual assessment rather than the automated detection of the software program according to previous reports [8,9,19,20,21]. Our reasoning was based on our experience that the automated detection can lead to erroneously short LDT, specifically for milking observations with bimodality. This is consistent with the recommendations given in the VaDia Suite User Manual which states: “The automatic function must be regarded to be of assistance for the manual adjustment. Results from the automatic splitting must always be checked before assessing vacuum conditions” (version 3.7, Biocontrol, Rakkestad, Norway). We believe that veterinarians and milk quality consultants will make best use of the data obtained with the VaDia recorder when the automatic detection is supplemented by visual assessment. In a recent study, Holst et al. [43] investigated differences in mouthpiece chamber vacuum and its cyclic fluctuations between milking liners with different liner shape (i.e., round vs. triangular) and type of cluster ventilation (i.e., claw vs. mouthpiece). They found higher mouthpiece chamber vacuum in triangular liners and the highest amplitude of fluctuation in the triangular liners with mouthpiece chamber ventilation [43]. These differences likely will be reflected on vacuum recordings using the VaDia device. Future studies to better understand how these differences influence the ability to visually assess the presence of bimodality would be beneficial.

The VaDia device only measured vacuum in 1 of the 4 short milk tubes and 2 of the 4 mouthpiece chambers for each cluster. Conversely, the Lactocorder device measured milk flow rate at the udder level. Since considerable variation in milk flow rate between quarters can exist [29], this could be viewed a limitation of the study.

As indicated by the discrepancies between the average claw vacuum values that were assessed during the milking center evaluations (Table 1) and the teat end vacuum documented from observational milkings (average cyclic vacuum fluctuations, Table 3), we observed a vacuum loss with the use of the Lactocorder as previously described [6]. This may have had an influence on our outcome measurements, namely bimodality and STC. However, our results are comparable with those of previous studies investigating bimodality [1,9] or STC [40,44]. We therefore believe that if such an influence had existed, it would have been minor.

The variability in bimodal milk ejection may be attributable to differences in premilking udder stimulation (i.e., extrinsic factors) and discrepancies in physiological requirements among cows (i.e., biological variation). To a smaller extent, differences in milking equipment may explain part of the variability in bimodality observed among different farms. Previous works showed that preparation lag time (i.e., time interval between first tactile stimulus and attachment of the milking unit) is a key risk factor of bimodality [22,45,46]. In the New York study [22], the investigators reported that the frequencies of bimodal milk flow curves in cows who received a preparation lag time of 0, 30, and 90 s, respectively, were 21, 14, and 7%. Further, in a recent study we showed that cows who received a short stimulation time (i.e., 7 s) had higher odds of bimodality as compared to animals who received a stimulation duration of 16 s [23] indicating that stimulation time (i.e., duration of tactile teat stimulation) has an effect on the risk of bimodality. For our study herds average preparation lag times and stimulation times ranged between 57–127 s and 3–6 s, respectively. This suggests that the main risk for bimodality was inadequate stimulation times (farms 1–5) and insufficient preparation lag time (farm 4). Current industry recommendations suggest a minimum stimulation time of 10 to 20 s and a preparation lag time of 90 s [47,48,49]. This may be impractical in many parlor systems and difficult to achieve through manual forestripping or wiping of teats alone. Future research should identify opportunities to accommodate the cows’ physiological requirements while improving parlor efficiency. This could be accomplished through combining manual and automated stimulation using inherent features such as the pulsator system.

Previous studies have identified breed [41], stage of lactation [1], the degree of udder filling [50], and chronic mastitis [11] as risk factor of bimodality indicating biological variation among cows with different characteristics. Several researchers suggested accommodating discrepancies in physiological requirements among cows through application of individual premilking stimulation regimens [45,51]. Future work is needed to further our understanding about these risk factors and whether addressing differences in physiological requirements of premilking stimulation can enhance milk production and udder health.

## 5. Conclusions

Our results show that BIM_VA_ and LDT can be used to estimate bimodal milk ejection in dairy cows and that the VaDia vacuum recorder can be used to assess milk flow curve dynamics during milking center evaluations. The association between BIM_LA_ and milk yield detected in this study suggests that bimodality is a useful measure to monitor milking performance on dairy operations. Future research is needed to identify risk factors of bimodality and investigate whether mitigation of bimodality through for example application of individual premilking stimulation regimens can enhance productivity and udder health of dairy cows. Last, we observed no association of BIM_VA_, BIM_LA_, and LDT on STC. We believe that unmeasured confounding factors such as teat traits hamper the suitability of bimodality as a measure to monitor STC. Further research to better understand the relationship between milk flow curve dynamics and STC would be beneficial.

## Figures and Tables

**Table 1:** Parlor type, milking machine equipment and settings, average claw vacuum values during the peak milk flow rate period, pulsator settings, cluster remover milk flow thresholds, and milking routine timings of 5 New York dairy farms assessed during 8 milking center evaluations. Values in parenthesis represent results from the second visit and were provided only when they deviated from those of the first visit.

Item	Farm 1	Farm 2	Farm 3	Farm 4	Farm 5
Parlor type	2 × 16 stall parallel	2 × 18 stall parallel	2 × 20 stall parallel	100 stall rotary	2 × 18 stall parallel
Vacuum pump capacity (kW)	11.2	11.2	11.2	22.4	11.2
Milkline vacuum (kPa) ^1^	45.0	43.0	41.7	44.0	44.0
Milkline diameter (cm)	7.6	7.6	7.6	10.1	7.6
Milking liner shape	triangular	multi-sided concave	triangular	round	triangular
Milking liner short milk tube diameter (mm)	10	12	11	12	10
Cluster ventilation ^2^	MPC + claw	claw	SMT + claw	claw	MPC + claw
Average claw vacuum ^3^ (kPa)	41.7	38.9	40.6 (42.3)	37.6	42.0 (41.7)
Pulsation rate ^4^ (cyles/min)	62	60	60	60	66 (64)
Pulsation ratio ^4^	65:35	65:35	65:35	65:35	66:34
Pulsation phases ^4^					
a-phase (ms)	113	112	104 (110)	150 (169)	97 (164)
b-phase (ms)	510	531	553 (551)	493 (476)	503 (449)
c-phase (ms)	82	101	79 (78)	122 (127)	79 (81)
d-phase (ms)	260	253	264 (263)	235 (228)	229 (239)
Automatic cluster remover settings (kg/min)	1.1	1.6	1.1	1.5	1.2
Dip contact time ^5^ (s)	91	105	105 (111)	26 (36)	83 (77)
Stimulation time ^6^ (s)	3	3	3	6	5
Preparation lag time ^7^ (s)	102	100	118 (125)	57 (78)	127 (124)

^1^ Mean value of milkline vacuum was assessed with an electronic vacuum measuring device (VPR200, DeLaval International AB, Tumba, Sweden) for a period of ≥30 min. ^2^ MPC: mouthpiece chamber ventilation, SMT: short milk tube ventilation, claw: ventilation via air vent in claw piece. ^3^ Mean average claw vacuum during peak milk flow period calculated from 10 individual cow milking observations assessed with an electronic vacuum measuring device (VPR200, DeLaval International AB, Tumba, Sweden). ^4^ Pulsation settings assessed with the VPR200 (DeLaval International AB) during the peak milk flow period of 1 individual cow milking observation. ^5^ Dip contact time: time interval between pre-dip application and drying and cleaning of teats; mean values assessed from a minimum of 12 individual cow milking observations. ^6^ Stimulation time: time interval between start of first tactile stimulus and termination of tactile stimulation; farms 1, 2, 3, and 5, duration of manual forestripping; farm 4, sum of wiping duration at positions 1 and 2. ^7^ Preparation lag time: time interval between first tactile stimulus and attachment of the milking unit; mean values assessed from a minimum of 12 individual cow milking observations.

**Table 2:** Descriptive statistics of 241 individual cow milking observations from 5 New York dairy farms. Values presented as mean and standard deviation unless otherwise stated.

Item	Farm 1	Farm 2	Farm 3	Farm 4	Farm 5	Total
n	30	34	48	67	62	241
Parity (n, %)						
1st	22 (73)	4 (12)	16 (33)	25 (37)	37 (60)	104 (43)
2nd	4 (13)	12 (35)	20 (42)	22 (33)	17 (27)	75 (31)
≥3rd	4 (13)	18 (53)	12 (25)	20 (30)	8 (13)	62 (26)
Stage of lactation (DIM)	145 ± 94	150 ± 83	150 ± 115	151 ± 99	142 ± 91	148 ± 97
lnSCC ^1^	3.4 ± 1.2	3.7 ± 1.0	4.3 ± 1.4	4.0 ± 1.2	3.9 ± 1.3	3.9 ± 1.3
Milk yield ^2^ (kg)	14.6 ± 4.4	15.5 ± 4.9	14.8 ± 4.4	13.8 ± 4.0	15.2 ± 3.3	14.7 ± 4.1
Machine on time ^3^ (s)	272 ± 109	288 ± 81	319 ± 92	228 ± 46	266 ± 74	270 ± 84
Let down time ^3^ (s)	34 ± 19	28 ± 23	38 ± 22	31 ± 19	27 ± 18	32 ± 20
Overmilking ^3^ (s)	63 ± 75	47 ± 39	68 ± 45	32 ± 23	42 ± 33	48 ± 44
ACVF ^4^ (kPa)	35.7 ± 2.6	32.9 ± 2.3	36.8 ± 5.7	34.0 ± 2.4	36.2 ± 2.4	35.2 ± 3.6
MPCV ^5^ (kPa)	13.5 ± 7.9	16.9 ± 9.5	18.2 ± 8.5	14.5 ± 7.4	17.4 ± 8.6	16.3 ± 8.4
BIM_VA_ ^6^ (n, %)	7 (23)	7 (21)	14 (29)	19 (28)	5 (8)	52 (22)
BIM_LA_ ^7^ (n, %)	10 (33)	8 (24)	15 (31)	17 (25)	7 (11)	57 (24)
STC ^8^ (n, %)	15 (50)	12 (35)	30 (63)	38 (57)	32 (52)	127 (53)

^1^ Natural logarithm of SCC from last DHIA test date. ^2^ Milk yield per individual cow milking observation recorded with the Lactocorder (WMB AG, Balgach, Switzerland). ^3^ Machine on time, let down time, and overmilking period recorded with the VaDia vacuum recorder (Biocontrol, Rakkestad, Norway) and assessed with the VaDia Suite software program (Biocontrol). ^4^ Average cyclic vacuum fluctuations (assessed for 10 pulsation cycles 60 s after the start of the peak milk flow period) recorded with the VaDia vacuum recorders (Biocontrol) and assessed with the VaDia Suite software program (Biocontrol). ^5^ Average mouthpiece chamber vacuum recorded with the VaDia vacuum recorders (Biocontrol) and assessed with the VaDia Suite software program (Biocontrol). ^6^ Presence of bimodality recorded with the VaDia vacuum recorders (Biocontrol) and visually assessed with the adjunct software program (VaDia Suite, Biocontrol); bimodality was present when mouthpiece chamber vacuum and teat end vacuum decreased after the start of milking but then increased markedly according to Erskine et al. [8]. ^7^ Presence of a bimodal milk flow curve recorded with the Lactocorder (WMB AG) and automatically assessed with the adjunct software program (LactoPro, WMB AG). ^8^ Presence of machine milking-induced short-term changes to the teat tissue condition assessed through visual inspection and palpation according to Hillerton et al. [31].

**Table 3:** Amount of bimodality assessed with the VaDia vacuum recorder (Biocontrol, Rakkestad, Norway) and Lactocorder (WMB AG, Balgach, Switzerland) from 241 individual cow milking observations.

VaDia	Lactocorder		Total
	Bimodality Present	Bimodality Absent	
Bimodality present	37	15	52
Bimodality absent	20	169	189
Total	57	184	241

**Table 4:** Descriptive statistics of milk yield, machine on time, let down time, overmilking period, average cyclic vacuum fluctuations (ACVF), average mouthpiece chamber vacuum (MPCV), and machine milking-induced short-term changes to the teat tissue condition (STC) of 241 individual cow milking observations stratified by the presence of bimodality as assessed with the VaDia vacuum recorder (BIM_VA_) and the Lactocorder (BIM_LA_), respectively. Values presented as mean and standard deviation unless otherwise stated.

Item	BIM_VA_		BIM_LA_	
	Present	Absent	Present	Absent
Milk yield (kg)	13.8 ± 3.6 ^a^	15.0 ± 4.2 ^a^	13.3 ± 4.2 ^a^	15.1 ± 4.0 ^b^
Machine on time (s)	256 ± 63 ^a^	274 ± 88 ^a^	254 ± 87 ^a^	275 ± 82 ^a^
Let down time (s)	59 ± 19 ^a^	24 ± 13 ^b^	50 ± 24 ^a^	26 ± 15 ^b^
Overmilking period (s)	48 ± 34 ^a^	48 ± 46 ^a^	46 ± 49 ^a^	49 ± 42 ^a^
ACVF (kPa)	35.1 ± 2.6 ^a^	35.2 ± 3.9 ^a^	35.7 ± 2.7 ^a^	35.1 ± 3.8 ^a^
MPCV (kPa)	13.4 ± 7.1 ^a^	17.0 ± 8.6 ^b^	14.4 ± 7.6 ^a^	16.8 ± 8.6 ^a^
STC (n, %)	27/52 (51.9) ^a^	100/189 (52.9) ^a^	27/57 (47.4) ^a^	100/184 (54.4) ^a^

^a,b^ Values with different superscript letters within a row of a variable differ at a level of *p* < 0.05 in Student’s *t*-test, Welch’s test (if Levene’s test indicated unequal variances), or Pearson chi-square test.

## Data Availability

The data presented in this study are available on request from the corresponding author.
